# Mood and mitochondria: how the endocannabinoid system fuels bipolar disorder

**DOI:** 10.1192/j.eurpsy.2026.10344

**Published:** 2026-07-31

**Authors:** H. Andreu Gracia, I. Pacchiarotti

**Affiliations:** Unidad de Trastornos Depresivos y Bipolares, Hospital Clínic de Barcelona, Barcelona, Spain

## Abstract

**Abstract:**

Bipolar disorder is increasingly recognised as a disorder involving impaired cellular energy regulation and neurobiological resilience. Mitochondrial dysfunction has been consistently associated with mood instability, cognitive impairment, and illness progression. In parallel, the endocannabinoid system has emerged as a key modulator of emotional regulation, synaptic plasticity, and intracellular energy pathways. This presentation will focus on current evidence linking alterations in endocannabinoid signalling to mitochondrial dysregulation, oxidative stress, and inflammatory mechanisms relevant to bipolar disorder. By integrating preclinical and clinical findings, the talk will highlight how the interaction between the endocannabinoid system and mitochondrial function may contribute to affective symptomatology and treatment response. These insights support a more integrative pathophysiological model and may inform the development of novel biomarkers and therapeutic strategies in bipolar disorder.

**Disclosure of Interest:**

None Declared

